# Diagnostic Performance of Dynamic Myocardial Perfusion Imaging Using Third-Generation Dual-Source Computed Tomography in Patients with Intermediate Pretest Probability of Coronary Artery Disease

**DOI:** 10.3390/jcdd12070264

**Published:** 2025-07-09

**Authors:** Sung Min Ko, Sung-Jin Cha, Hyunjung Kim, Pil-Hyun Jeon, Sang-Hyun Jeon, Sung Gyun Ahn, Jung-Woo Son

**Affiliations:** 1 Department of Radiology, Wonju Severance Christian Hospital, Yonsei University Wonju College of Medicine, 20 Ilsan-ro, Wonju 26426, Republic of Korea; cktjdwls9612a@gmail.com (S.-J.C.); radkhj@yonsei.ac.kr (H.K.); iromeo138@naver.com (P.-H.J.); leadledled@naver.com (S.-H.J.); 2 Department of Cardiology, Wonju Severance Christian Hospital, Yonsei University Wonju College of Medicine, 20 Ilsan-ro, Wonju 26426, Republic of Korea; sgahn@yonsei.ac.kr (S.G.A.); soneycar@gmail.com (J.-W.S.)

**Keywords:** computed tomography, coronary artery disease, myocardial perfusion, fractional flow reserve, myocardial ischemia

## Abstract

(1) Background: Our aim was to evaluate the diagnostic performance of combined coronary computed tomography angiography (CCTA) and dynamic CT myocardial perfusion imaging (CT-MPI) for detecting hemodynamically significant coronary artery disease (CAD) in patients with intermediate pretest probability. (2) Methods: Patients with an intermediate pretest probability of CAD were retrospectively enrolled. All patients underwent CCTA and dynamic CT-MPI using a third-generation dual-source CT scanner prior to invasive coronary angiography (ICA). Anatomically significant stenosis was defined as ≥50% luminal narrowing on both CCTA and ICA. Fractional flow reserve (FFR) was performed during ICA in selected cases. Hemodynamically significant CAD was defined per vessel as FFR ≤ 0.80, angiographic stenosis ≥70%, or having undergone revascularization. The diagnostic performance of CCTA alone and CCTA combined with CT-MPI was compared against this reference standard. (3) Results: Seventy-four patients (mean age, 66.8 ± 11.1 years; 59 men) were included. The median coronary calcium score was 508.5 Agatston units (interquartile range: 147–1173). ICA and CCTA detected anatomically significant stenoses in 137 (61.7%) and 146 (65.8%) coronary vessels, respectively, and in 62 (83.8%) and 71 (95.9%) patients, respectively. Hemodynamically significant stenosis was present in 56 patients (76%) and 99 vessels (45%). On a per-vessel basis, CCTA alone yielded a sensitivity of 96.7%, specificity of 60.3%, positive predictive value (PPV) of 64.4%, and negative predictive value (NPV) of 96.1%. Combined CCTA and CT-MPI demonstrated a sensitivity of 90.1%, specificity of 84.3%, PPV of 82.7%, and NPV of 91.1%. The area under the receiver operating characteristic curve improved from 0.787 (95% confidence interval: 0.73–0.84) for CCTA to 0.872 (95% confidence interval: 0.82–0.91) for the combined approach (*p* < 0.05). The median total radiation dose for both CCTA and CT-MPI was 8.05 mSv (interquartile range: 6.71–11.0). (4) Conclusions: In patients with intermediate pretest probability of CAD, combining CCTA with dynamic CT-MPI significantly enhances the diagnostic performance for identifying hemodynamically significant coronary stenosis compared to CCTA alone.

## 1. Introduction

Recent advances in computed tomography (CT) technology have established coronary CT angiography (CCTA) as a key noninvasive modality for evaluating coronary artery disease (CAD). CCTA provides direct anatomical assessment of coronary stenosis and plaque, and is widely used as a gatekeeping tool before invasive coronary angiography (ICA) [1,2]. The UK National Institute for Health and Care Excellence (NICE) guidelines recommend CCTA as the first-line diagnostic test for suspected CAD, regardless of pre-test probability [3]. Although CCTA is effective in detecting anatomical stenosis—particularly lesions with ≥50% luminal narrowing—it does not offer information on the functional significance of these lesions, such as their potential to induce myocardial ischemia [4]. To assess the functional impact of the detected coronary artery stenoses, additional diagnostic tests are necessary. Myocardial perfusion imaging (MPI) using single-photon emission computed tomography (SPECT), positron emission tomography (PET), or cardiovascular magnetic resonance (CMR) is commonly used to evaluate myocardial ischemia and viability [5,6]. More recently, CT-derived fractional flow reserve (CT-FFR) and CT myocardial perfusion imaging (CT-MPI) have been introduced to assess the hemodynamic relevance of coronary stenosis. These techniques have shown good diagnostic performance compared with invasive FFR [7].

Dynamic CT-MPI quantifies myocardial blood flow (MBF) by analyzing contrast enhancement kinetics during pharmacologic stress, similar to stress perfusion CMR [8]. Dynamic CT-MPI can be a useful tool in evaluating patients with significant coronary artery calcification (CAC), coronary stents, or other inconclusive tests suggestive of CAD [9,10,11,12]. However, further validation is required to define its role across different clinical scenarios. This study aimed to evaluate the diagnostic performance of combined CCTA and dynamic CT-MPI using third-generation dual-source CT for detecting hemodynamically significant coronary stenosis in patients with intermediate pre-test probability of CAD.

## 2. Materials and Methods

### 2.1. Patient Population

This retrospective study was approved by the Institutional Review Board, and the requirement for informed consent was waived. A total of 422 patients who underwent combined CCTA and dynamic CT-MPI between April 2020 and March 2022 were initially identified from our institutional database. Among these, 98 symptomatic patients aged ≥45 years with an intermediate pretest probability of obstructive CAD who also underwent invasive coronary angiography (ICA) were selected for analysis. The pretest probability of obstructive CAD was estimated using the 2019 European Society of Cardiology (ESC) web-based risk calculator, based on the CAD Consortium Clinical Model incorporating age, sex, symptom type, and cardiovascular risk factors [1]. Exclusion criteria were as follows: (1) Patients with echocardiography and CT findings of myocardial infarction (MI); (2) patients with echocardiography and CT findings accompanied by cardiomyopathy or other significant cardiovascular disease; and (3) patients with impaired image quality of dynamic CT-MPI or CCTA. A total of 24 patients were excluded due to prior myocardial infarction (*n* = 10), hypertrophic cardiomyopathy (*n* = 4), severe aortic regurgitation (*n* = 2), severe mitral regurgitation (*n* = 1), and non-diagnostic image quality on CT-MPI (*n* = 5) or CCTA (*n* = 2). The final study population included 74 patients.

### 2.2. Dynamic CT-MPI and CCTA Protocol

**Figure 1:**
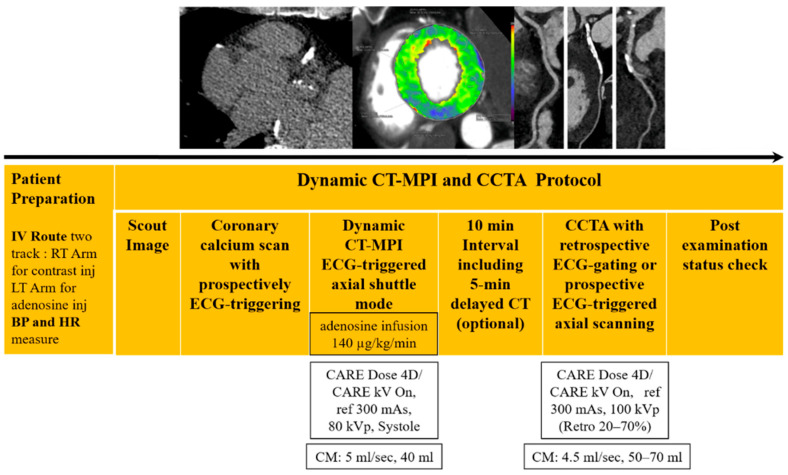
Integrating anatomical and functional assessment of coronary artery disease using dynamic CT-MPI and CCTA. After patient preparation and monitoring, non-contrast prospective electrocardiography-guided axial CT for calcium scoring was obtained. Stress CT-MPI is acquired in ECG-triggered axial shuttle mode via intravenous adenosine infusion lasting at least 3 min. An optional 5 min delayed CT scan can be added while waiting 10 min after stress CT-MPI. CCTA is performed using prospective ECG-triggered or retrospective ECG-gated scanning with ECG pulsing based on HR. CT-MPI = computed tomography-myocardial perfusion imaging, CCTA = coronary computed tomography angiography, IV = intravenous, BP = blood pressure, HR = heart rate, ECG = electrocardiography, CM = contrast media.

All patients underwent a standardized imaging protocol consisting of coronary calcium scoring, dynamic stress CT-MPI, and CCTA (Figure 1), performed on a third-generation dual-source CT scanner (SOMATOM Force, Siemens Healthineers, Forchheim, Germany). Patients were instructed to abstain from caffeine for 24 h prior to the examination and to avoid beta-blockers and theophylline on the day of the scan. The scan range for dynamic CT-MPI was planned based on the coronary calcium scoring images to ensure full coverage of the left ventricle (LV) and all coronary arteries. Dynamic stress CT-MPI was performed using adenosine triphosphate (ATP) infusion at 140 µg/kg/min over 3 min. Contrast medium (40 mL of iopromide, 370 mg iodine/mL, Ultravist 370^®^, Bayer AG, 13353 Berline, Germany) was injected at 5 mL/s, followed by a 30 mL saline flush using a dual-head power injector (Dual Shot GX7; Nemoto Kyorindo, Tokyo, Japan). Image acquisition commenced 4 s after the start of contrast injection. Dynamic perfusion was acquired during the end-systolic phase using shuttle mode (coverage: 105 mm) over a fixed duration of 32 s, capturing 10–15 image phases depending on heart rate (HR). Acquisition parameters included the following: collimation, 96 × 0.6 mm; rotation time, 250 ms; CARE kV with a reference of 80 kVp; and CARE Dose 4D with an effective tube current of 300 mAs. Baseline HR was measured prior to ATP infusion, and peak HR was recorded 3 min after infusion. HR increment was defined as the difference between peak and baseline HR. Ten minutes after dynamic CT-MPI, 0.6 mg of sublingual nitroglycerin was administered prior to CCTA. No beta-blockers were given for HR control. CCTA was performed using prospective ECG-triggered axial scanning for patients with HR < 65 bpm and retrospective ECG-gated acquisition with ECG pulsing for HR ≥ 65 bpm. A tri-phasic injection protocol was used at 4.5 mL/s. First, 0.9 mL/kg of iopromide (Ultravist 370^®^) was administered, followed by 45 mL of a 70:30 mixture of contrast and saline, and then 30 mL of saline. All injections were delivered using the same dual-head injector system.

### 2.3. CT Data Reconstruction and Image Post-Processing

Dynamic CT-MPI images were reconstructed in the axial plane using a slice thickness of 3 mm with a 2 mm overlap and a medium-sharp convolution kernel (Qr40). Image analysis was performed using commercially available software (Volume Perfusion CT Body; Siemens) on the postprocessing workstation (Syngo.via VB10, Siemens Healthineers, Forchheim, Germany) MBF was quantified using a dedicated parametric deconvolution technique based on a two-compartment model representing intra- and extravascular spaces, applied to time–attenuation curves [13,14]. MBF was calculated as the ratio of the maximum slope of the fitted myocardial enhancement curve to the peak of the arterial input function. The data were then processed using prototype software (Cardiac Functional Analysis 3.0.1; Siemens Healthineers) that automatically segmented the LV based on a standard heart model and generated 17-segment polar maps of subendocardial MBF distribution. CCTA images were reconstructed using a medium–soft convolution kernel (Bv40) and a third-generation iterative reconstruction algorithm (ADMIRE, strength level 3; Siemens Healthineers). Data sets were transferred to offline workstations (Vitrea version 7.12; Vital Images, Minnetonka, MN, USA and syngo.via; Siemens Healthineers) for post-processing. Optimal mid-diastolic and end-systolic phases were selected for further analysis.

### 2.4. Image Analysis

To minimize interpretation bias, image analysis was performed under strict blinding protocols. Two radiologists (with 22 and 4 years of experience in cardiac imaging) initially interpreted CCTA images in consensus, without access to clinical data, dynamic CT-MPI, ICA, or FFR results. One month later, the same radiologists reviewed CT-MPI images in consensus, independently of the CCTA findings, clinical data, ICA, or FFR results. After both assessments, CCTA and CT-MPI results were compared side by side solely for assigning myocardial perfusion territories based on coronary anatomy.

The coronary arteries were evaluated according to a 3-vessel and 16-segment coronary artery model modified from the American Heart Association (AHA) classification [15]. Each segment was classified as having either non-significant (<50% reduction in lumen diameter) or significant (≥50% reduction in lumen diameter) stenosis. Segments deemed non-diagnostic due to motion artifacts or severe coronary artery calcification (CAC) were interpreted as having significant stenosis to avoid an underestimation of disease burden. A coronary vessel was considered as having a significant lesion if it contained at least one segment with ≥50% stenosis or any non-diagnostic segment [16]. 

Myocardial perfusion defects involving two or more contiguous segments on CT-MPI were considered indicative of ischemia. Based on the interpretation of available CCTA and CT-MPI images, the presence of hemodynamically significant CAD was determined per vessel territory. If CCTA and CT-MPI findings were discrepant, then myocardial perfusion overruled CCTA stenosis severity, unless CT-MPI image quality was compromised. Vessel-level disease classification was determined by the most severely affected branch within the respective perfusion territory. The quantification of MBF was performed by placing round regions of interest (ROIs) measuring 1–2 cm^2^ within each of the 16 myocardial segments (excluding the apex) on the short-axis MBF map. ROIs were positioned at least 2 mm from both endocardial and epicardial borders to avoid contamination [17,18]. Hyperemic myocardial regions were excluded due to the potential overestimation of perfusion. Myocardial ischemia was defined as an absolute MBF value < 100 mL/100 mL/min [19].

### 2.5. ICA and FFR

All patients underwent ICA using a standard 6F technique on a dedicated cardiac catheterization system (Allura Xper FD10, Philips Healthcare, Amsterdam, The Netherlands). ICA was performed at a median of 6 days (interquartile range [IQR]: 1–30 days) following combined dynamic CT-MPI and CCTA. Quantitative stenosis assessment was conducted using a dedicated analysis platform (CAAS; Pie Medical Imaging, Maastricht, The Netherlands) with automated caliper and scaling functions. Coronary artery segmentation matched the CCTA analysis, and stenosis ≥ 50% in luminal diameter was considered anatomically obstructive.

FFR was selectively performed at the discretion of the interventional cardiologist, typically in patients with intermediate stenosis (50–70%), non-specific or atypical symptoms, or lesions involving major epicardial arteries where revascularization decisions were uncertain. This reflects real-world practice in Korea, where FFR is reimbursed but not routinely applied in all cases. FFR measurements were obtained following the induction of maximal hyperemia, either via an intravenous infusion of adenosine (140 μg/kg/min) or intracoronary injection of nicorandil (2 mg). A lesion was considered functionally non-significant if the FFR value exceeded 0.80. All ICA and FFR data were analyzed by a single cardiologist with 20 years of experience in interventional cardiology, who was blinded to both CCTA and CT-MPI findings.

### 2.6. Radiation Dose

The effective radiation dose for coronary calcium scoring, CT-MPI, and CCTA was calculated in all patients. For CT, the dose-length product (DLP) was converted to mSv by multiplying it by a conversion coefficient (κ = 0.014 mSv·mGy^−1^·cm^−1^) [20].

### 2.7. Statistical Analysis

For each coronary artery, the presence of hemodynamically significant stenosis is indicated by FFR ≤ 0.80, invasive angiographic severity ≥ 70%, or by using a revascularization procedure as reference. Continuous variables are presented as mean ± SD or median and interquartile range (IQR). Categorical variables are given as frequencies and percentages. The diagnostic performance of CCTA alone and in combination with dynamic CT-MPI was evaluated on a per-vessel basis, using the reference standard described above. Diagnostic metrics included sensitivity, specificity, positive predictive value (PPV), negative predictive value (NPV), and accuracy, each reported with 95% confidence intervals (CIs). Comparisons of sensitivity, specificity, and accuracy between CCTA alone and CCTA combined with CT-MPI were performed using the McNemar test. Receiver operating characteristic (ROC) curves were generated, and the area under the ROC curve (AUC) with 95% CI was calculated for both CCTA and CCTA + CT-MPI. All statistical analyses were conducted using SPSS software (version 25.0; IBM Corp., Armonk, NY, USA). AUC comparisons were performed using MedCalc (version 19.5.3; MedCalc Software Ltd., Ostend, Belgium). A two-sided *p*-value of <0.05 was considered statistically significant.

## 3. Results

### 3.1. Patient Characteristics

The clinical characteristics of the 74 patients included in the study are summarized in Table 1. The mean age was 66.8 ± 11.1 years (range, 45–92 years), and 59 patients (80%) were male. Typical angina symptoms were reported in 59 patients (80%), and all patients (100%) were on cardiac medications. Prior to undergoing CCTA, 51 patients (69%) had evidence of CAC on preceding chest (*n* = 30) or abdominal (*n* = 21) CT scans, performed at a median interval of 55 days (IQR: 33–76 days). The median coronary calcium score (CCS) was 508.5 Agatston units (IQR: 147–1173), with 41 patients (55%) exhibiting severe CAC (CCS > 400 Agatston units).

Anatomically significant coronary stenoses were identified in 137 vessels (62.7%) in 62 patients (83.8%) by ICA, and in 146 vessels (65.8%) in 71 patients (95.9%) by CCTA. Among these, 12 patients (16.2%) had single-vessel disease, 25 (33.8%) had two-vessel disease, and 25 (33.8%) had anatomically significant three-vessel disease, as confirmed by ICA. No cases of left main coronary artery disease were observed. Hemodynamically significant stenosis was present in 56 patients (76%) and 99 vessels (45%), as determined by the composite reference standard. CT-MPI revealed 116 ischemic vascular territories in 62 patients. The median dose-length products (DLP) for CCTA and CT-MPI were 241 mGy·cm (IQR: 193–315) and 331 mGy·cm (IQR: 315–452), respectively. The median total effective radiation dose for the combined CCTA and CT-MPI examination was 8.05 (IQR:6.71–11.0) mSv (Table 2).

### 3.2. Diagnostic Performance of CCTA and CT-MPI

**Figure 2:**
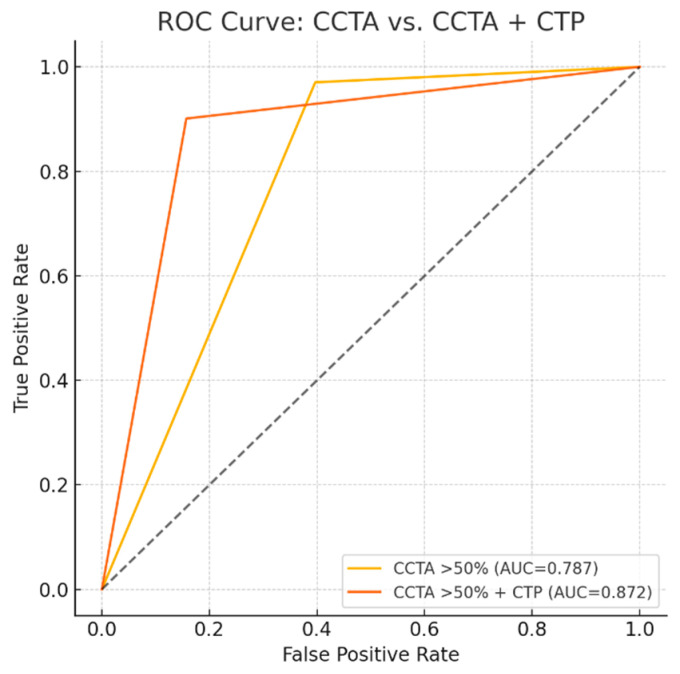
Receiver-operating characteristic curves for the detection of hemodynamically significant coronary artery disease. Per-vascular territory analysis shows that the AUC increases significantly from 0.787 with CCTA alone to 0.872 with the addition of CT-MPI (*p* < 0.05). AUC: area under the curve; CT-MPI: computed tomography-myocardial perfusion imaging; CCTA: coronary computed tomography angiography.

**Figure 3:**
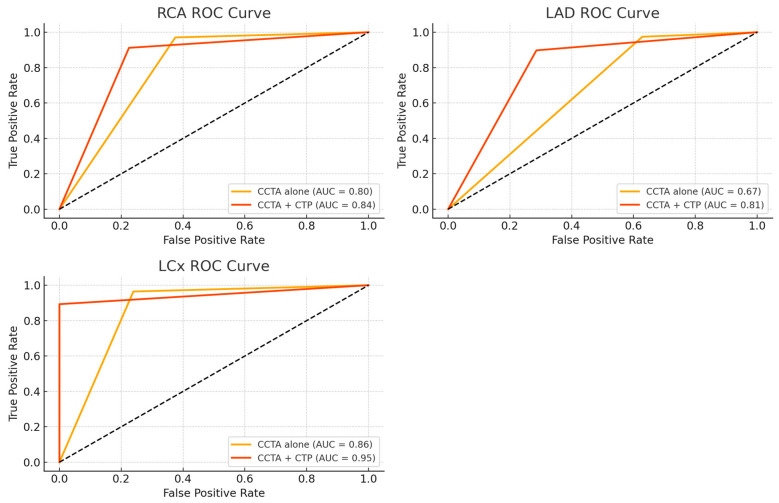
Vessel-specific receiver-operating characteristic (ROC) curves for detecting hemodynamically significant coronary artery disease. ROC curves comparing CCTA alone and combined CCTA with CT-MPI for detecting hemodynamically significant coronary stenosis in the RCA, LAD, and LCx. The addition of CT-MPI increased AUC from 0.799 to 0.844 in the RCA (*p* = 0.523), from 0.674 to 0.811 in the LAD (*p* = 0.097), and from 0.863 to 0.945 in the LCx (*p* = 0.142), demonstrating consistent improvement across all territories. AUC: area under the curve; CT-MPI: computed tomography-myocardial perfusion imaging; CCTA: coronary computed tomography angiography; RCA: right coronary artery; LAD: left anterior descending coronary artery; LCx: left circumflex artery.

Upon per-vessel analysis (Table 3), the sensitivity, specificity, PPV, NPV, and accuracy for detecting hemodynamically significant coronary stenosis were 96.7%, 60.3%, 64.4%, 96.1%, and 75.8%, respectively, for CCTA alone (stenosis ≥ 50%), and 90.1%, 84.3%, 82.7%, 91.1%, and 86.9%, respectively, for combined CCTA and CT-MPI. The addition of CT-MPI significantly improved specificity (84.3% vs. 60.3%, *p* < 0.001) and overall accuracy (86.9% vs. 75.8%, *p* = 0.004) compared to CCTA alone. There was no significant difference in sensitivity between the two methods (90.1% vs. 96.7%, *p* = 0.11). The area under the ROC curve (AUC) increased significantly from 0.787 (95% CI: 0.73–0.84) to 0.872 (95% CI: 0.82–0.91) with the addition of CT-MPI (*p* < 0.05) (Figure 2). Vessel-specific ROC analysis showed consistent improvement in diagnostic performance with the addition of CT-MPI across all territories. AUC increased from 0.799 to 0.844 in the right coronary artery (*p* = 0.523), from 0.674 to 0.811 in the left anterior coronary artery (*p* = 0.097), and from 0.863 to 0.945 in the left circumflex artery (*p* = 0.142), although statistical significance was not reached in individual vessels (Figure 3). In a subgroup analysis using only lesions with invasive FFR (≤0.80) as the reference standard (Table 4), the addition of CT-MPI significantly increased specificity (60% vs. 0%, *p* < 0.001) and diagnostic accuracy (74% vs. 35%, *p* < 0.001), confirming the main results.

**Figure 4:**
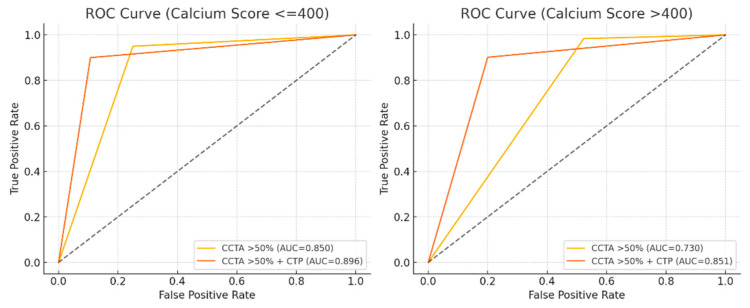
Receiver-operating characteristic (ROC) curves for the detection of hemodynamically significant coronary artery disease, stratified by coronary calcium score. Among patients with CCS ≤ 400 (**left panel**), the AUC increases from 0.850 with CCTA alone to 0.896 with combined CCTA and CT-MPI (*p* = 0.09). In patients with CCS > 400 (**right panel**), the AUC significantly improves from 0.73 to 0.85 with the addition of CT-MPI (*p* < 0.001). AUC: area under the curve; CCS: coronary calcium score; CT-MPI: computed tomography-myocardial perfusion imaging; CCTA: coronary computed tomography angiography.

**Figure 5:**
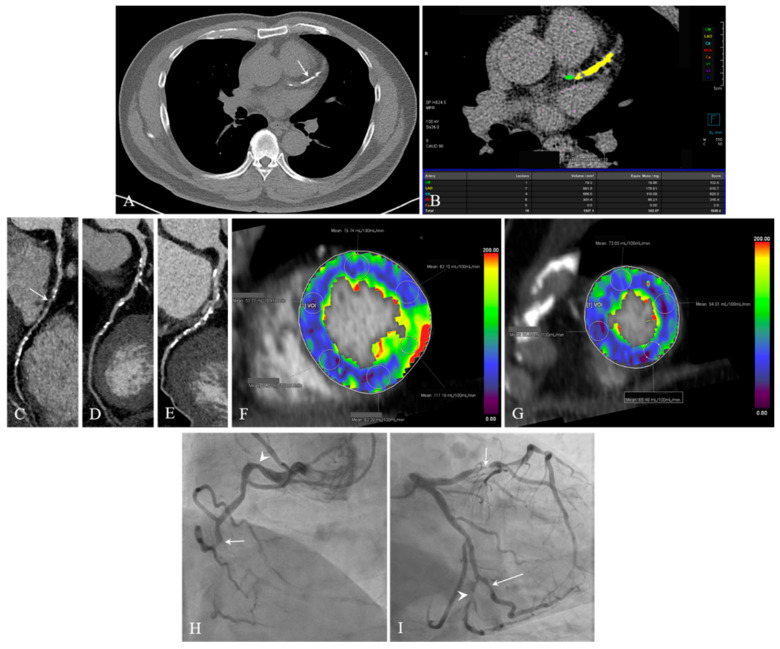
Images of a 60-year-old man with atypical chest pain. (**A**) Low-dose chest CT taken 45 days before CCTA shows severe CAC in the LAD (arrow). (**B**) Coronary calcium scan shows a high coronary calcium score of 1848.4 AU. (**C**–**E**) Curved MPR CCTA images of RCA (**C**), LAD (**D**), and LCx (**E**) show severe CAC. A severe stenosis with mixed calcified and noncalcified plaques is seen in the mid segment of the RCA (**C**, arrow). (**F**,**G**) CT-MPIs show an overall decrease in MBF from the mid- (**F**) to apical (**G**) segments, excluding the mid-inferolateral segment. (**H**,**I**) ICA identifies chronic total occlusion of the mid-RCA (**H**, arrow), significant stenosis (80–90% lumen reduction) in the proximal to mid-LAD (**I**, short arrow) and distal LCx (**I**, arrowhead), and significant stenosis (50–60% lumen reduction) in the proximal RCA (**H**, arrowhead) and secondary OM branch (I, long arrow). CT = computed tomography; RCA: right coronary artery, LAD = left anterior descending coronary artery, LCx = left circumflex artery, OM = obtuse marginal, AU = Agatston unit, MBF = myocardial blood flow, ICA = invasive coronary angiography, CT-MPI = computed tomography-myocardial perfusion imaging, CCTA = coronary computed tomography angiography, MPR = multiplanar reformation.

**Figure 6:**
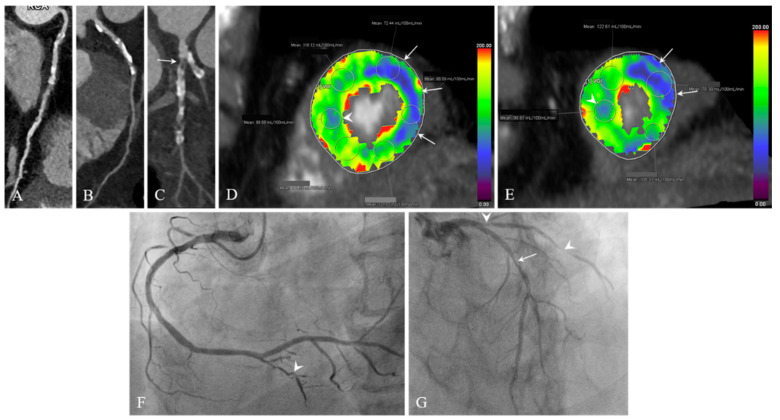
Images of a 74-year-old man with typical angina. (**A**–**C**) Curved MPR CCTA images of RCA (**A**), LAD (**B**), and LCx (**C**) show severe CAC. Severe stenosis with mixed calcified and noncalcified plaques is seen in the proximal portion of the LCx (**C**, arrow). (**D**,**E**) CT-MPIs show decreased MBF in the mid- to apical lateral (arrows), mid-anteroseptal (**D**, arrowhead), and apical septal (**E**, arrowhead) segments, with a greater decrease in the mid- to apical lateral segments. (**F**,**G**) ICA confirms significant stenosis (80% lumen reduction) in the small PDA (**F**, arrowhead) and proximal and distal LCxs (**G**, arrowheads), and significant stenosis (65% lumen reduction) in the proximal to middle LAD (**G**, arrow). The invasive FFR for the middle LAD is 0.70. CT-MPI = computed tomography-myocardial perfusion imaging, CCTA = coronary computed tomography angiography, MPR = multiplanar reformation, MBF = myocardial blood flow, ICA = invasive coronary angiography, FFR = fractional flow reserve, PDA = posterior descending artery, RCA = right coronary artery, LAD = left anterior descending coronary artery, LCx = left circumflex artery.

Among patients with no to moderate CAC (CCS ≤ 400 Agatston units; *n* = 32), the addition of CT-MPI significantly improved specificity (from 75.0% to 89.3%, *p* = 0.013) while preserving high sensitivity and accuracy. Although the AUC increased from 0.85 (95% CI: 0.777–0.912) to 0.896 (95% CI: 0.832–0.957), the difference was not statistically significant (*p* = 0.09). In contrast, among patients with severe CAC (CCS > 400 Agatston units; *n* = 42), CCTA + CT-MPI significantly improved specificity, PPV, and accuracy (*p* < 0.05), while sensitivity and NPV declined (Table 5). Notably, the AUC significantly increased from 0.73 (95% CI: 0.67–0.79) to 0.85 (95% CI: 0.785–0.907, *p* < 0.001) (Figure 4). Among 67 severely calcified coronary arteries classified as anatomically significant on CCTA, 40 were determined to have hemodynamically significant stenosis and 27 were functionally insignificant. CCTA alone identified all 67 arteries as functionally significant, yielding a sensitivity of 100% (95% CI: 94.6–100%), specificity of 0%, PPV of 59.7% (95% CI: 44.7–70.6%), and overall accuracy of 59.7% (95% CI: 44.7–70.6%). In contrast, combined CCTA and CT-MPI identified 46 arteries as functionally significant (35 true positives, 11 false positives) and 21 arteries as functionally insignificant (16 true negatives, 5 false negatives) (Figure 5 and Figure 6). This yielded a sensitivity of 87.5% (95% CI: 77.3–97.8%), specificity of 59.3% (95% CI: 40.7–77.8%), PPV of 76.1% (95% CI: 63.8–88.4%), NPV of 76.2% (95% CI: 58.0–94.4%), and diagnostic accuracy of 76.1% (95% CI: 65.9–86.3%). CCTA + CT-MPI significantly improved specificity and accuracy (*p* < 0.05).

### 3.3. Quantitative MBF Analysis

All dynamic CT-MPI examinations were of diagnostically acceptable image quality. Using an absolute MBF threshold of <100 mL/100 mL/min to define ischemia, 447 out of 1184 myocardial segments (37.8%) were classified as ischemic. The median MBF in territories supplied by vessels with functionally significant CAD was 87.7 mL/100 mL/min (IQR, 79.2–94.2), which was significantly lower than in remote territories without significant CAD, where the median MBF was 153.3 mL/100 mL/min (IQR, 130.6–175.3) (*p* < 0.001).

## 4. Discussion

The main findings of this study are as follows: (1) the addition of dynamic CT-MPI to CCTA significantly improved diagnostic accuracy for detecting hemodynamically significant CAD in patients with intermediate pretest probability, and (2) absolute MBF values effectively discriminated myocardial territories supplied by functionally significant versus non-significant vessels. CAD remains a leading global cause of morbidity and mortality [21]. CCTA provides high diagnostic accuracy and excellent NPV in patients with intermediate pretest probability of CAD, enabling the avoidance of unnecessary ICA [22]. However, in patients with extensive CAC, CCTA is associated with reduced specificity and increased false-positive findings, often leading to an overestimation of stenosis severity [23]. In such cases, further functional assessment—either through noninvasive imaging or invasive FFR—is essential to guide appropriate revascularization decisions [24]. Recent updates in CAD-RADS 2.0 recommend adjunctive functional imaging, such as CT-FFR or dynamic CT-MPI, for patients with 50–90% stenosis (CAD-RADS categories 3 and 4A) or proximal coronary artery narrowing ≥40%, including in the presence of high-risk plaque features (CAD-RADS category 2) [25]. Our findings support this recommendation, particularly in patients with high coronary calcium burden where anatomic evaluation alone may be insufficient.

This study focuses on patients with an intermediate pretest probability of CAD and offers important insights into the diagnostic utility of combining CCTA with dynamic CT-MPI, particularly in clinically challenging scenarios [11]. A key feature of the cohort was the inclusion of 42 patients with severe CAC (CCS > 400) and 67 severely calcified coronary arteries, underscoring the real-world applicability of this approach. Although combined CCTA and CT-MPI demonstrated slightly lower sensitivity (90.1%) than CCTA alone (96.7%), both approaches exhibited high sensitivity, supporting their utility in identifying hemodynamically significant lesions. Notably, the addition of CT-MPI significantly improved specificity (84.3% vs. 60.3%) and diagnostic accuracy (86.9% vs. 75.8%), along with a significant increase in AUC (0.87 vs. 0.79, *p* < 0.05). Although the vessel-specific AUC improvements with CT-MPI were not statistically significant, the consistent trend across all three coronary territories supports its incremental diagnostic value. The observed benefit was most pronounced in the left anterior descending coronary artery. Subgroup analysis using a 400 Agatston unit threshold revealed that in patients with lower calcium scores (≤400), CT-MPI significantly improved specificity (from 75.0% to 89.3%, *p* = 0.013), with a non-significant trend toward improved AUC (from 0.850 to 0.896, *p* = 0.09). In contrast, among patients with higher calcium burden (>400 AU), the addition of CT-MPI significantly enhanced specificity, PPV, accuracy (*p* < 0.05), and AUC (from 0.730 to 0.850, *p* = 0.042). These findings underscore the value of integrating functional imaging with anatomic assessment to improve diagnostic performance in patients with extensive CAC. However, the increase in false-negative cases with combined CCTA and CT-MPI likely reflects a degree of anatomical–functional mismatch or limitations in the sensitivity of CT-MPI. Despite the proven diagnostic accuracy of CT-MPI, its broader clinical adoption remains limited due to practical challenges in standardization. Variability in scanner platforms, contrast protocols, acquisition timing, and pharmacologic stress agents contributes to significant heterogeneity in scan time, radiation dose, and overall cost. Collaborative efforts are needed to develop standardized acquisition and interpretation protocols to support consistent implementation in routine practice [7,8,14,25].

It is important to note that the prevalence of hemodynamically significant CAD in this study was relatively high (76% per patient, 45% per vessel), exceeding that of typical diagnostic cohorts (25–35%). This elevated prevalence may influence PPV, NPV, accuracy, and AUC, yet it reflects the clinical reality of our institution, where patients are referred for CCTA and CT-MPI based on intermediate pretest probability, prior imaging findings, or persistent risk factors. As such, the reported diagnostic performance should be interpreted in the context of a high-risk, preselected population. Our study is distinct in that it reflects a real-world, intermediate-risk population with severe CAC, where CCTA interpretation is often limited. Due to restricted use of invasive FFR, hemodynamic significance was defined pragmatically based on ≥70% stenosis or clinical decisions leading to PCI or CABG. Furthermore, in a setting where CT-FFR is not available, this study demonstrates the practical utility of dynamic CT-MPI as a functional alternative. These findings may serve as a reference for future research in similar clinical environments.

In this study, a threshold of 100 mL/min/100 mL was used to define myocardial ischemia, consistent with prior research [18,19]. The median MBF in territories supplied by vessels with functionally significant CAD was significantly lower (87.7 mL/100 mL/min) compared to remote territories (153.3 mL/100 mL/min, *p* < 0.001). This clear distinction supports the diagnostic value of dynamic CT-MPI in differentiating ischemic from non-ischemic myocardium. These findings underscore the complementary role of CT-MPI to CCTA by providing quantitative functional assessment, thereby improving the diagnostic accuracy and clinical relevance of coronary CT evaluation. Although confounding factors such as heart rate, body mass index, or CAC may influence absolute MBF values, our per-territory analysis design allowed within-subject comparison, which mitigated inter-individual variability. Future studies incorporating multivariate models may further investigate the role of MBF as an independent predictor of ischemia.

This study has several limitations. First, it is a retrospective, single-center study with a relatively small sample size (*n* = 74), which may limit generalizability. Second, although all patients were categorized as having an intermediate pretest probability of obstructive CAD based on the 2019 ESC guidelines [1], the prevalence of functionally significant CAD in our cohort was relatively high, potentially introducing spectrum bias. Third, invasive FFR was not performed in all patients. The primary findings were also confirmed in a subgroup analysis restricted to lesions with invasive FFR ≤ 0.80 as the reference standard, demonstrating consistent improvements in diagnostic performance with the addition of CT-MPI. A composite reference standard—FFR ≤ 0.80, ICA stenosis ≥ 70%, or revascularization—was adopted to reflect real-world practice, where treatment decisions are often based on angiographic severity and clinical context, particularly in cases of multivessel or high-grade disease. A potential source of selection bias is the institutional diagnostic pathway: most patients were elderly males with poorly controlled risk factors and pre-existing coronary calcification noted on prior chest or abdominal CT. Due to the high calcium burden, CCTA alone was often insufficient for definitive assessment. Given the lack of access to CT-FFR and the limited routine use of invasive FFR in Korea, dynamic CT-MPI was clinically selected to complement CCTA in this population. Lastly, ischemia was defined using an absolute MBF threshold (<100 mL/100 mL/min), which may differ from relative MBF techniques used in other protocols [26]. These limitations suggest that, while this study offers meaningful insight into the combined application of CCTA and dynamic CT-MPI for CAD assessment, further validation is required. Prospective, multicenter studies with larger cohorts, complete invasive FFR data, and standardized MBF measurement protocols are essential to confirm these findings.

## 5. Conclusions

In conclusion, among patients with an intermediate pre-test probability of CAD—particularly those with severe CAC—the addition of dynamic CT-MPI to CCTA enhances diagnostic accuracy for identifying hemodynamically significant coronary stenosis. The incremental value of dynamic CT-MPI warrants further evaluation in larger, multinational trials.

## Figures and Tables

**Table 1:** Baseline characteristics of the study cohort (*n* = 74).

Gender (M:F)	59:15 (80%:20%)
Age (years)	66.8 ± 11.1
Heart Rate (bpm)	64.5 ± 11.7
BSA (m^2^)	1.7 ± 0.2
Risk Factors	
HTN	57 (77.0%)
DM	34 (45.9%)
Hyperlipidemia	27 (36.5%)
Smoking	32 (43.2%)
CAD Family History	2 (2.7%)
COPD	70 (94.6%)
Heart Medication	74 (100%)
Chest Pain	Typical angina, 59 (80%) Atypical angina, 15 (20%)
Intermediate Pretest Probability of CAD (15–85%)	50.5 ± 19.0
Image Quality	
Good to excellent	70 (94.6%)
Poor but acceptable	4 (5.4%)
Coronary Calcium Score (AU)	508.5 (IQR: 1173–147)
Classification of Coronary Calcium Score	
None to moderate: 0–400 AU	32 (43.2%)
Severe: >400 AU	42 (56.8%)

BSA = body surface area, COPD = chronic obstructive pulmonary disease, HTN = hypertension, DM = diabetes mellitus, CAD = coronary artery disease, IQR = interquartile range; AU = Agatston units.

**Table 2:** Radiation dose summary (n = 74).

Parameter	CCTA	CT-MPI	CCTA + CT-MPI
CTDIvol (mGy)	15.5 (IQR:12.6–20.0)	31.0 (IQR:25.3–42.4)	–
DLP (mGy·cm)	241 (IQR:193–315)	331 (IQR:315–452)	–
Effective Dose (mSv)	3.37 (IQR:2.70–4.41)	4.63 (IQR:3.74–6.33)	8.05 (IQR:6.71–11.01)

CCTA: coronary computed tomography angiography; CT-MPI: computed tomography-myocardial perfusion imaging; DLP: dose-length product; CTDIvol: computed tomography dose index volume.

**Table 3:** Per-vessel diagnostic accuracy of CCTA and CCTA plus CT-MPI using a composite reference standard (n = 222).

	Sensitivity	Specificity	PPV	NPV	Accuracy	TP	TN	FP	FN
**CCTA stenosis** **of >50%**	96.7 (90.7–99.1)	60.3 (51.4–68.6)	64.4 (56.1–72.0)	96.1 (89.0–98.9)	75.8 (69.5–81.4)	87	73	48	3
**CCTA stenosis** **of >50% plus** **CT-MPI**	90.1 (82.7–94.5)	84.3 (76.8–89.7)	82.7 (74.6–88.7)	91.1 (84.3–95.1)	86.9 (81.8–91.1)	91	102	19	10

PPV = positive predictive value, NPV = negative predictive value, TP = true positive, TN = true negative, FP = false positive, FN = false negative, CT-MPI = computed tomography-myocardial perfusion imaging, CCTA = coronary computed tomography angiography.

**Table 4:** Per-vessel diagnostic accuracy of CCTA and CCTA plus CT-MPI using invasive FFR (n = 23) as the reference standard.

	Sensitivity	Specificity	PPV	NPV	Accuracy	TP	TN	FP	FN
**CCTA stenosis** **of >50%**	100 (68.0–100)	0	35.0 (19.0–55.0)	0	35.0 (19.0–55.0)	8	0	15	0
**CCTA stenosis** **of >50% plus** **CT-MPI**	100 (68.0–100)	60.0 (36.0–80.0)	57.0 (33.0–79.0)	100 (70.0–100)	74.0 (54.0–87.0)	8	9	6	0

PPV: positive predictive value; NPV: negative predictive value; TP; true positive; TN: true negative; FP: false positive; FN: false negative; CT-MPI: computed tomography-myocardial perfusion imaging; CCTA: coronary computed tomography angiography.

**Table 5:** Per-vessel diagnostic performance of CCTA and combined CCTA with CT-MPI stratified by coronary calcium score.

	Sensitivity	Specificity	PPV	NPV	Accuracy	TP	TN	FP	FN
**CCS** ** ≤ ** **400**									
**CCTA stenosis** **of >50%**	95.0 (83.5–98.6)	75.0 (62.3–84.5)	73.1 (59.7–83.2)	95.5 (84.9–98.7)	83.3 (74.6–89.5)	38	42	14	2
**CCTA stenosis** **of >50% plus** **CT-MPI**	90.0 (76.9–96.0)	89.3 (78.5–95.0)	85.7 (72.2–93.3)	92.6 (82.4–100)	89.6 (81.9–94.2)	36	50	6	4
**CCS > 400**									
**CCTA stenosis** **of >50%**	98.4 (91.3–99.7)	47.7 (36.0–59.6)	63.8 (53.8–72.8))	96.9 (84.3–99.4)	77.2 (63.8–79.3)	60	31	34	1
**CCTA stenosis** **of >50% plus** **CT-MPI**	90.2 (80.2–95.4)	80.0 (68.7–87.9)	80.9 (70.0–88.5)	89.7 (79.2–95.2)	84.9 (77.6–90.1)	55	52	13	6

PPV: positive predictive value; NPV: negative predictive value; TP; true positive; TN: true negative; FP: false positive; FN: false negative; CT-MPI: computed tomography-myocardial perfusion imaging; CCTA: coronary computed tomography angiography.

## Data Availability

The original contributions presented in this study are included in the article. Further inquiries can be directed to the corresponding author.

## Abbreviations

CAD	obstructive coronary artery disease
CT-MPI	computed tomography-myocardial perfusion imaging
CT-FFR	computed tomography-fractional flow reserve
CCTA	coronary computed tomography angiography
CAC	coronary artery calcification
CCS	coronary calcium score
AUC	area under the receiver-operating characteristic curve
MBF	myocardial blood flow
PCI	percutaneous coronary intervention
CABG	coronary artery bypass grafting
MI	myocardial infarction
ICA	invasive coronary angiography
